# Supplementary figures and images for: Phenotypic and genotypic analysis of drug resistance in M. tuberculosis isolates in Gansu, China
Source: PLoS One. 2024 Sep 27;19(9):e0311042. doi: 10.1371/journal.pone.0311042 (PMC11432870; doi:10.1371/journal.pone.0311042)

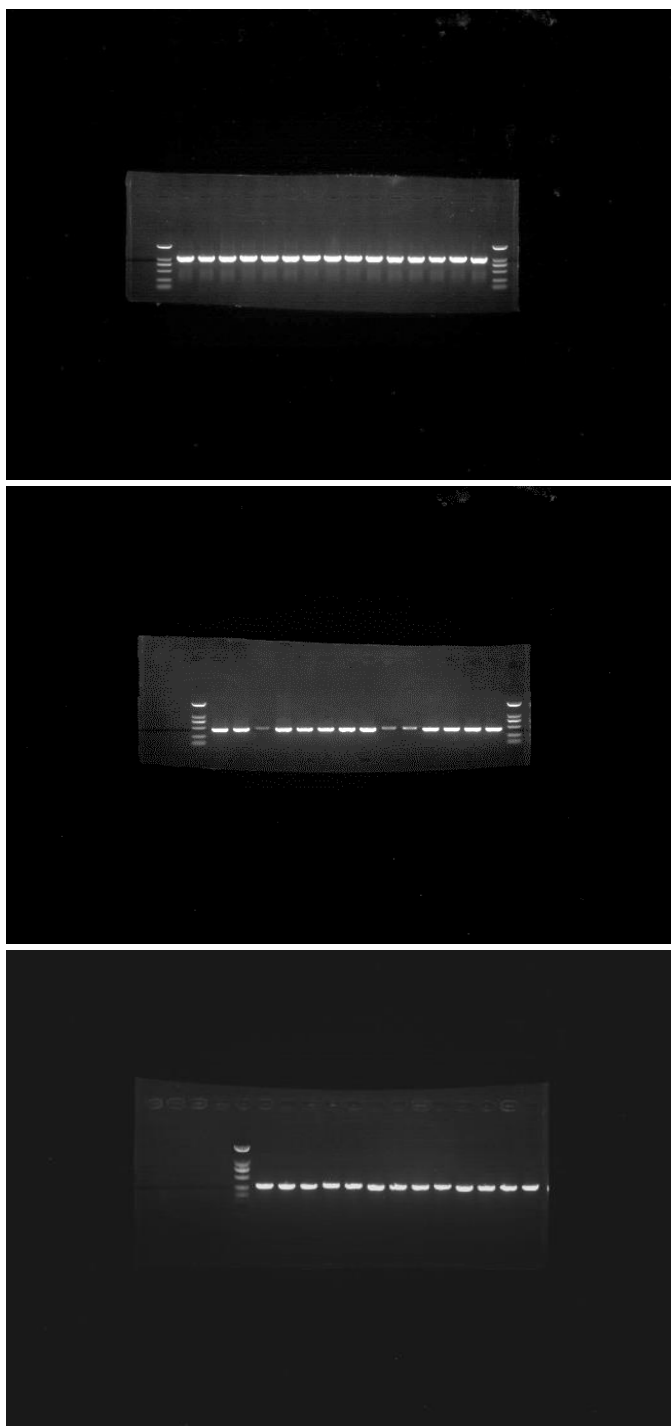

S11-Fig

Supplement: S1 Raw image — (PDF) [file pone.0311042.s002.pdf]
