# Supplementary material for: Phenotypic and genotypic analysis of drug resistance in M. tuberculosis isolates in Gansu, China
Source: PLoS One. 2024 Sep 27;19(9):e0311042. doi: 10.1371/journal.pone.0311042 (PMC11432870; doi:10.1371/journal.pone.0311042)
Supplement: Attachment — Submitted filename: Response to Reviewers.docx [file pone.0311042.s004.docx]

**Responses to the editor and reviewers comments**

***Editor (Comments for the Author):***

Thank you for submitting your manuscript to PLOS ONE. After careful consideration, we feel that it has merit but does not fully meet PLOS ONE’s publication criteria as it currently stands. Therefore, we invite you to submit a revised version of the manuscript that addresses the points raised during the review process. Please submit your revised manuscript by Sep 02 2024 11:59PM.

Response: Thank you for your consideration and work. The review’s comments are all valuable and very helpful for revising and improving our paper. We have revised the manuscript carefully and responded to the reviewers’ comments point-by-point.

***Reviewer #3 (Comments for the Author):***

*Q1. The author need to separately analyze pulmonary and extra pulmonary isolates as the site of infections are different and they cannot be mixed up and shown as they all are drug resistant. Show the results separately for pulmonary and extra-pulmonary isolates.*

Response: Many thanks to the reviewer for the comments. In the diagnosis of tuberculosis, sputum is typically utilized as the primary sample. This is due to the propensity of tuberculosis patients to expel the pathogen from pulmonary lesions into the sputum through coughing and other mechanisms. Consequently, the presence of *Mycobacterium tuberculosis* and other pertinent evidence can be more directly identified through sputum analysis. However, not all manifestations of tuberculosis are confined to the lungs. Tuberculosis can affect various parts of the body, including the meninges (tuberculous meningitis), bones (bone tuberculosis), and intestines (intestinal tuberculosis). Consequently, diagnostic samples from the affected sites, such as cerebrospinal fluid, bone tissue, and intestinal tissue, can provide a more accurate assessment of the disease's extent and severity. Therefore, reliance solely on sputum samples is insufficient for a comprehensive diagnosis. Moreover, alternative sample types should be employed for patients who are unable to produce sputum or experience difficulty in doing so, to facilitate accurate diagnosis. Utilizing diverse sample types can offer a multifaceted basis for tuberculosis diagnosis. The etiological agent of tuberculosis, irrespective of the infection site, is typically *Mycobacterium tuberculosis.* Bedaquiline is an antimicrobial agent specifically targeting *Mycobacterium tuberculosis*. We did not conduct separate analyses of the samples from the lungs and other anatomical regions, as our experimental focus was on *Mycobacterium tuberculosis*, which exhibits consistent characteristics across the various sample sources. We sincerely hope that this response will satisfy the reviewers.

*Q2. The authors need to show comparison of Bedaquiline mutations identified in their study with other WGS studies as Bedaquiline mutations have been also identified in other WGS studies. Please add this information is table2 showing what is unique and common mutations as compared to other studies.*

Response: Many thanks to the reviewer for the comments. We reviewed the literature and added in Table 2 the mutation sites of previously identified Bedaquiline resistance genes. We also demonstrate that the mutation sites of the Bedaquiline resistance gene identified in this paper are inconsistent with previously identified mutations (page 9, lines 246-249).
